# SuperPATH Minimally Invasive Approach to Total Hip Arthroplasty of Femoral Neck Fractures in the Elderly: Preliminary Clinical Results

**DOI:** 10.1111/os.12584

**Published:** 2019-12-29

**Authors:** Xiao‐dong Wang, Hai Lan, Zheng‐xia Hu, Kai‐nan Li, Zheng‐hao Wang, Jin Luo, Xu‐dong Long

**Affiliations:** ^1^ Zunyi Medical University Zunyi China; ^2^ Department of Orthopaedics Affiliated Hospital of Chengdu University Chengdu China

**Keywords:** Femoral neck fractures, Minimally invasive, SuperPATH approach, Total hip arthroplasty

## Abstract

**Objective:**

To investigate the clinical efficacy and advantages of the SuperPATH minimally invasive approach to total hip arthroplasty in the treatment of femoral neck fractures in the elderly.

**Methods:**

From January 2016 to September 2018, 110 cases of elderly patients with femoral neck fractures were included in the present study. According to the method of operation, the patients were divided into two groups for comparison. There were 55 cases of the SuperPATH minimally invasive approach to total hip arthroplasty and 55 cases with the conventional posterolateral approach to total hip arthroplasty. The operation time, the length of incision, the amount of operative blood loss, the hospitalization time, and the hospitalization cost were compared between the two groups. The position of total hip prosthesis was observed during the follow‐up period. All patients were evaluated for the degree of hip joint pain and the function of the hip joint using the visual analog score (VAS) and the Harris score at 1 week, 1 month, 3 months, 6 months, and 12 months after the operation.

**Results:**

All patients were followed up for at least 12 months. The operation time was 108.58 ± 15.87 min in the SuperPATH group and 102.51 ± 19.61 min in the conventional group. The length of incision was 6.65 ± 1.53 cm in the SuperPATH group and 17.08 ± 1.40 cm in the conventional group. The amount of operative blood loss was 147.51 ± 28.84 mL in the SuperPATH group and 170.22 ± 25.34 mL in the conventional group. The hospitalization time was 10.05 ± 2.52 days in the SuperPATH group and 13.36 ± 3.39 days in the conventional group. The hospitalization cost was 6871.78 ± 141.63 dollars in the SuperPATH group and 7791.09 ± 184.88 dollars in the conventional group. Compared with the conventional group, the SuperPATH group had shorter incision length, less blood loss, shorter hospitalization time, and lower hospitalization cost. There was significant difference between the two groups (*P* < 0.05). In the two groups, there were no complications such as infection, lower extremity venous thrombosis, prosthesis loosening, periprosthetic fracture, and dislocation during the follow‐up period. The VAS score was 4.45 ± 0.94 in the SuperPATH group and 4.89 ± 0.79 in the conventional group at 1 week after the operation. There was significant difference between the two groups (*P* < 0.05). The Harris score was 75.36 ± 3.36 and 80.25 ± 3.09 in the SuperPATH group and 68.80 ± 3.25 and 77.35 ± 3.77 in the conventional group at 1 week and 1 month after the operation, respectively. There was significant difference between the two groups (*P* < 0.05). In the analysis of the operation time, the VAS score at 1 month, 3 months, 6 months, and 12 months after the operation, and the Harris score at 3 months, 6 months, and 12 months after surgery, there was no significant difference between the two groups (*P* > 0.05).

**Conclusion:**

The SuperPATH minimally invasive approach to total hip arthroplasty is an ideal method for the treatment of femoral neck fractures in the elderly. This method has the advantages of the relatively simple operation, short incision, less blood loss, and less trauma. The patients had short hospitalization times, low hospitalization costs, and good recovery of hip joint function.

## Introduction

With the intensification of the aging of the world population, the incidence of hip diseases in the elderly is also increasing. Hip fractures are especially common, with 20%–30% of individuals with hip fractures dying within 1 year^1^, ^2^. Femoral neck fractures are the most common type of hip fracture, accounting for 48%–54% of hip fractures. Approximately 90% of the patients with femoral neck fractures are over 50 years old, and the incidence is increasing year by year^3^. Most of the elderly patients with femoral neck fractures have osteoporosis, with falling or twisting often resulting in femoral neck fractures. Complications such as fracture nonunion and avascular necrosis of the femoral head often occur after femoral neck fractures^4^. The Garden classification of femoral neck fractures divides these fractures into four types^5^. Type I are incomplete fractures,with the trabeculae below the femoral neck intact. This type includes the so‐called “abduction and intercalation fractures”. Type II are complete fractures, but there is no displacement. Type III are complete fractures with partial displacement. On the X‐ray film of this type of fracture, the distal end of the fracture can be seen to move up with external rotation, the femoral head is often backward, and there is still partial contact at the end of the fracture. Type IV are complete fractures with complete displacement. The X‐ray film of this type of fracture showed that there is no contact at the broken end of the fracture, but the relative relationship between the femoral head and the acetabulum is normal. The treatment of elderly patients with femoral neck fractures can be divided into conservative treatment and surgical treatment. In elderly patients with femoral neck fractures, long‐term bed rest is likely to lead to pulmonary infection, pressure sores, deep venous thrombosis, and other complications. Therefore, most scholars believe that, in the absence of absolute surgical contraindications, surgical treatment is conducive to the recovery of patients^6^. At present, commonly used surgical methods are cannulated screw internal fixation and total hip arthroplasty. For elderly patients with femoral neck fractures aged between 65 and 75 years, those with Garden types III and IV are able to tolerate surgery. At present, the first choice of surgical treatment is total hip arthroplasty. Total hip arthroplasty for the treatment of femoral neck fractures in the elderly, through the reconstruction of hip joint function, can not only reduce the long‐term pain, but also improve the mobility of the hip joint^7^. It restores the ability of elderly patients with femoral neck fractures to walk normally and improves the quality of life of the patients.

Total hip arthroplasty has become an effective and important approach for the treatment of hip diseases since it was first used in clinic in the 1920s. Total hip arthroplasty can relieve joint pain, correct deformity, restore and improve the motor function of the hip joint, and improve the quality of life of patients^8^. There are many surgical approaches for total hip arthroplasty, among which the posterolateral approach is widely used in clinic. However, this approach involves removing the posterior external rotation muscle groups and the joint capsule of the hip joint, which affects the stability of the joint and increases the risk of dislocation^9^. At the same time, as a result of muscle injury, increased postoperative bleeding aggravated the degree of pain of the patients, so that the rehabilitation time was prolonged^10^. With the improvement of minimally invasive surgical techniques and increased research into rapid rehabilitation, the application of minimally invasive surgery has become more commonplace for total hip arthroplasty^11^. The SuperPATH approach is a minimally invasive total hip arthroplasty approach that has been used in recent years. The SuperPATH approach was initiated and reported by Professor James Chow in the USA^12^. The incision is only 6–8 cm, and is made through the muscle space between the piriformis and the gluteus minimus without cutting off the external circumflex muscle groups of the hip. Total hip arthroplasty is accomplished by means of the acetabular transdermal channel and special tools^13^. Using this surgical approach, almost all the muscle function around the hip joint and the complete joint capsule are preserved, which is beneficial to the rapid recovery of the patients. It overcomes the shortcomings of traditional total hip arthroplasty, including slow recovery and long‐term bed rest. It demonstrates excellent clinical application value and is receiving increasing attention. The SuperPATH minimally invasive approach to total hip arthroplasty for the treatment of femoral neck fractures in the elderly helps to lessen surgical trauma, reduce hospital stay, and improve hip function. In addition, it can effectively reduce the hospitalization costs of patients and reduce the burden of patients. This surgical approach can promote the rapid recovery of patients after operation.

The purpose of this study is as follows: (i) to compare the clinical efficacy of the SuperPATH minimally invasive approach to total hip arthroplasty and the conventional posterolateral approach to artificial total hip arthroplasty in the treatment of femoral neck fractures in the elderly; and (ii) to explore the advantages of the SuperPATH minimally invasive approach to total hip arthroplasty in the treatment of femoral neck fractures in the elderly.

## Materials and Methods

### *Inclusion and Exclusion Criteria*

#### *Inclusion Criteria*

The inclusion criteria are: (i) the age of the patient was 65 to 75 years old, unilateral closed femoral neck fractures diagnosed with X‐ray or CT, and the Garden classification was type III or IV; (ii) the surgical approach was either the SuperPATH minimally invasive approach to total hip arthroplasty or the conventional posterolateral approach to total hip arthroplasty for femoral neck fractures; (iii) the major evaluation indicators included the operation time, the length of incision, the amount of operative blood loss, the hospitalization time, the hospitalization cost, follow up, the visual analog score (VAS) (1 week, 1 month, 3 months, 6 months, and 12 months after operation), and the Harris score (1 week, 1 month, 3 months, 6 months, and 12 months after the operation); and (iv) this study is a prospective case‐control study.

#### *Exclusion Criteria*

Exclusion criteria were: (i) the patients had hip joint disease, severe hip joint anatomical deformity, and hip joint dysfunction before fracture; (ii) the patients had a history of hip surgery before fracture; (iii) the patients had a history of severe heart, kidney, or liver complications or tumors; (iv) the patients had a history of thrombosis, abnormal coagulation function, use of anticoagulants, or risk of bleeding before fracture; (v) the patients had serious medical diseases that affect the recovery of joint function, and neuromuscular or skeletal muscle diseases that adversely affect gait or weight‐bearing; (vi) body mass index (BMI) >30 kg/m^2^; and (vii) the follow‐up time was less than 12 months.

### *General Information of Participants*

From January 2016 to September 2018, 110 cases of elderly patients with femoral neck fractures were included in the study. According to the method of operation, the patients were divided into two groups for comparison. There were 55 cases of the SuperPATH minimally invasive approach to total hip arthroplasty and 55 cases of the conventional posterolateral approach to total hip arthroplasty. The study protocol was approved by our Institutional Review Board. All patients had provided informed consent to the treatment plan, the operation plan, and the rehabilitation and follow‐up process.

Surgical internal fixation implants: For the SuperPATH group, the SuperPATH total hip prosthesis system made by MicroPort Orthopedics incorporated (USA) was selected. For the conventional group, the total hip prosthesis system produced by DePuy Medical Technology Company (Switzerland) was selected.

### *Preoperative Preparation*

After hospitalization, the patients were treated according to the damage control strategy. Skin traction was used to treat femoral neck fractures. To determine the type and displacement of the fracture, lateral X‐rays and CT three‐dimensional reconstruction images of the pelvis and the injured hip joint were examined preoperatively. At the same time, color doppler ultrasonography and D‐dimer examination were performed to observe the presence of venous thrombosis in lower extremities. Preoperative treatment mainly concerns the original medical diseases, and involves, for example, blood pressure control, blood glucose control, pain management, nutritional support, and correction of electrolyte disorders. At the same time, the health status of patients and whether they could tolerate surgery were comprehensively evaluated. Antibiotics were administered 30 min before the operation to prevent infection.

### *Surgical Methods*

#### *SuperPATH Minimally Invasive Approach to Total Hip Arthroplasty* ^14^

##### *Patient Preparation and Disinfection*

All the patients in the group underwent surgery under general anesthesia. After successful anesthesia, the baffle was placed on the anterior and posterior sides of the pubic symphysis, and the patient was kept in the lateral position. Make the hip tilt later, with the hip flexion 45° and the injured lower limb internal rotation at 10° to 15°, so that the greater trochanter was facing up. The area was disinfected and sterile sheets were laid out.

##### *Stripping of Soft Tissue*

A 6 to 8 cm incision was made from the tip of the greater trochanter on the injured side to the proximal end of the femur. The incision ended at the level of the fascia on the surface of the gluteus maximus muscle. The gluteus maximus muscle was separated with two pterygoid tip retractors and a Cobb retractor was placed below the gluteus medius muscle. It was then replaced with a blunt Hohmann retractor which was placed in the space between the gluteus medius muscle and the gluteus minor muscle to protect the gluteus medius muscle.

##### *Exposure of Articular Capsule*

The assistant abducted and rotated the hip joint. A Cobb retractor was placed behind the gap between the piriformis tendon and the gluteus minimus. Then it was replaced by a blunt Hohmann retractor, which was placed between the posterior articular capsule and the external rotator muscle to expose the articular capsule. The joint capsule was cut from the saddle of the femoral neck and extended to the proximal end to the acetabulum. The joint capsule was marked so that it could be identified at the time of suture. The pear fossa, the apex of the greater trochanter of the femur, and the neck of the anterior femur were exposed.

##### *Enlargement and Take Shape Femoral Medullary Cavity*

The assistant pressed the knee joint to rotate the affected limb slightly inward. The sellar part of the femoral neck was exposed, an open reamer was used to enter the femoral medullary cavity through the piriform fossa, and the bone chisel expanded the proximal opening. With the appropriate size of round calcar punch and impact handle expansion slot, the appropriate medullary cavity file was selected to shape the medullary cavity, and the curette was used to treat the proximal and middle segment of the femur. The medullary cavity file was inserted and the handle removed.

##### *Remove Femoral Head*

A pendulum saw was used to amputate the femoral neck along the top of the medullary file. A threaded needle was inserted into the femoral head, and with its leverage force, the femoral head was rotated to a large internal position, and then a threaded needle was inserted into another hard part of the femoral head. By rotating, the femoral head was removed and the diameter of the femoral head was measured.

##### *Acetabular Preparation*

The Zelpi retractor was placed under the periosteum of the proximal acetabular margin of the incision and the Romanelli retractor was placed in the distal joint to remove all the remaining soft tissue on the acetabulum and acetabular lip.

##### *Establishment of Percutaneous Approach and Grinded Acetabulum*

The blunt trocar and casing were placed through the deflector. A horizontal incision of approximately 1 cm was made at the intersection of the trocar and the thigh and it was inserted deep along the posterior 1 to 2 cm of the femur until the blunt trocar and the cannula could be seen through the main incision. The aiming handle and the blunt trocar were removed, only indwelling casing. The acetabular file handle was used to place the appropriate size acetabular file. The rod of the acetabular file was inserted through the casing and matched with the acetabular file in situ. The acetabular file grinded acetabulum from small to large.

##### *Place Acetabular Cup*

After grinding the acetabulum, the appropriate acetabular cup and lining were placed. Acetabular screw fixation was used to increase the stability.

##### *Reset Test Mold and Remove*

The appropriate femoral head and neck was selected, the femoral neck was installed in the pulp file, the femoral head was installed in the acetabular cup, with the opening facing up and back. The blunt trocar was inserted into the top of the medullary file to reset the hip joint. Check the tightness of the joint and the size of the sphere to ensure that the range of motion of the hip joint is good and there is no prolapse in the test mold. The tip of the blunt trocar was inserted into the upper hole of the test mold femoral neck, and the two instruments were separated and confronted with each other, so that the test mold femoral neck and medullary cavity file were separated and dislocated. The femoral neck, femoral head, and femoral bone marrow cavity file were removed.

##### *Assembly Prosthesis*

After the molds were removed, the artificial femoral head, the artificial femoral neck, and the femoral handle prosthesis of the same type were implanted and installed firmly. Finally, the hip joint was reset and the range of motion of the hip joint was examined.

##### *Close the Wound*

The area around the incision was rinsed and approximately 50 mL tranexamic acid solution was injected to reduce blood oozing. No active bleeding was found and the joint capsule was sutured. According to the situation during the operation, it was determined whether the drainage tube should be placed or not. After counting the instruments and dressings, the wound was closed layer by layer and the sterile dressings were bandaged. The surgery was the complete.

**Figure 1:**
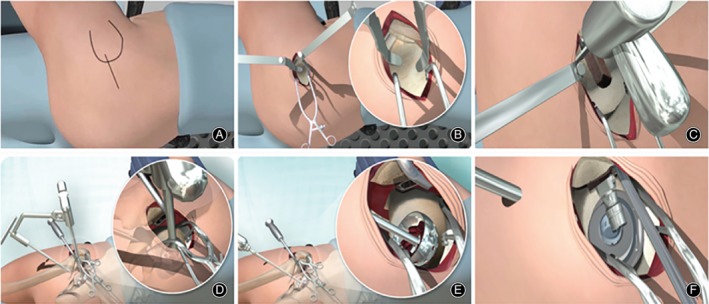
Surgical diagrams of the SuperPATH minimally invasive approach total hip arthroplasty: (A) Surgical incision and approach; (B) exposure of articular capsule; (C) amputation of the femoral neck; (D) establishment of percutaneous approach; (E) ground acetabulum; and (F) assembly prosthesis.

Surgical diagrams of the SuperPATH minimally invasive approach to total hip arthroplasty are shown in Fig. 1 (provided by MicroPort Orthopedics, USA).

#### *Conventional Posterolateral Approach to Total Hip Arthroplasty*

##### *Patient Preparation and Disinfection*

Compared with the SuperPATH group, all the patients in the conventional group were anesthetized in the same way. The patients took the lateral recumbent position, the pelvis vertical operating table and fixed. The area was disinfected and sterile sheets were laid out

##### *Stripping of Soft Tissue*

Modified Gibson incision was used. The subcutaneous incision was made in front of the posterior superior iliac spine and a 15 to 20 cm arc incision was made from the greater trochanter to the distal end of the femoral shaft. By cutting open the broad fascia and separating the gluteus maximus muscle along the direction of muscle fibers, the external circumflex muscle groups can be exposed.

##### *Exposure and Incision of Articular Capsule*

The assistant rotationed hip rotation. The tendons of piriformis muscle, superior and inferior muscle, and internal obturator muscle were cut off. The quadratus femoris muscle was protected and the proximal part cut off if necessary. The joint capsule was exposed and the joint capsule and acetabular glenoid lip was removed.

##### *Dislocation of Hip Joint*

The upper and lower parts of the articular capsule were loosened as fully as possible to remove all the osteophytes at the posterior edge of the acetabulum that hindered the dislocation of the femoral head. Flexion, adduction, and slight internal rotation of the hip joint can cause posterior dislocation.

##### *Remove Femoral Head*

An electric knife or bone knife can be used to mark the osteotomy line at the predetermined osteotomy position of the femoral neck. A swing saw was used to amputate the neck of the femur. Separate any soft tissue attached to the femoral head and remove the femoral head.

##### *Acetabular Preparation*

The femur was pulled forward and medial and rotated gently to expose the acetabulum. All residual soft tissue was removed from the acetabulum and the acetabular lip. The round ligament of the femoral head was excised before curettage of any residual soft tissue in the occipital region.

##### *Ground Acetabulum*

The femur was fully pulled forward so that the acetabular file was unobstructed from the front into the acetabulum, and the acetabular file ground the acetabulum from small to large.

##### *Place Acetabular Cup*

After grinding the acetabulum, the appropriate acetabular cup and lining were placed. Acetabular screw fixation was used to increase the stability.

##### *Enlargement and Shaping of Femoral Medullary Cavity*

The acetabular lining test mold was placed into the acetabular cup and then the side of the femur was processed. The medullary cavity was drilled and reamed into the femoral medullary cavity through the piriform fossa, and the bone chisel expanded the proximal opening. The appropriate pulp cavity file was selected for medullary cavity shaping, and the proximal and middle femur was treated with curettage. The appropriate medullary file was inserted, and the handle removed.

##### *Reset Test Mold and Remove*

The appropriate femoral head and neck were selected and the hip joint was reset. The tightness of the joint and the size of the sphere were checked to ensure that the range of motion of the hip joint was good and there was no prolapse in the test mold. If the stability of the hip joint were acceptable, flexion and internal rotation of the hip joint were undertaken to make it dislocated. The test film was removed, ready to install the prosthesis.

##### *Assembly Prosthesis*

After the molds were removed, the same type of prosthesis and lining were implanted and firmly installed. Finally, the hip joint was reset and the range of motion of the hip joint was checked.

##### *Close the Wound*

The area around the incision was rinsed and approximately 50 mL tranexamic acid solution was injected to reduce blood oozing. No active bleeding was found and the joint capsule was sutured. According to the situation during the operation, whether or not the drainage tube should be placed was determined. The severed piriformis muscle was sutured *in situ*. After counting the instruments and dressings, the wound was closed layer by layer and the sterile dressings were bandaged. The surgery was the complete.

### *Postoperative Treatment and Follow‐up*

#### *Postoperative Treatment*

After surgery, the patients were treated according to the total hip arthroplasty ERAS strategy. Following the operation, the patients were given symptomatic treatment, nutritional support, and strategies for pain management, infection prevention, blood management, and electrolyte balance. At the same time, attention was paid to the prevention of complications, such as venous thrombosis and pneumonia of lower extremities, as well as the treatment of original medical diseases and anti‐osteoporosis. The patients’ blood routine, electrolytes, and inflammatory reaction index were reexamined. X‐ray films of the pelvis and injured hip joints were reexamined. The drainage tube was retained for 24 to 48 h, and the sutures were removed in the two groups after 2 weeks.

#### *Postural Restriction*

In the SuperPATH group, there was no special postural restriction. In the conventional group, special restricted positions, such as abduction and external rotation, were necessary after the operation. The injured lateral position was not recommended in the conventional group. When in the healthy lateral position, it is necessary to clip the triangular pillow between the two legs to raise and abduct the affected limb. The hip flexion should not exceed 90° within 3 months. Patients were not allowed to bend the hip and rotate the affected limb at the same time, and were not to sit on a low stool or cross legs within half a year.

#### *Functional Exercise*

According to the actual situation of patients, early functional exercise was carried out under the guidance of rehabilitation doctors. On the day after the operation, the patients in the two groups were advised to undertake active hip exercise and lower limb strength training. Depending on the patient's general condition, the patient could be allowed to carry out weight‐bearing walking with the help of a walker after 1 day. Based on the specific rehabilitation of the patients, walking alone was allowed within 1 month, and completely independent walking was allowed after 1 month. When walking, those in the conventional group were advised to keep their toes forward and to follow the requirements of posture restrictions.

#### *Follow‐up*

The postoperative follow‐up plan was similar between the two groups. All patients were followed up regularly after the operation for at least 12 months. X‐ray films of the pelvis and the injured hip joint were reexamined. During the follow‐up period, the deformities of the affected limb were observed. The position, the abduction angle, and the anteversion angle of total hip prosthesis were observed. At the same time, the degree of hip joint pain and functional recovery on the injured side were observed and recorded.

### *Observation Indicators*

#### *The Operation Time*

The operation time was recorded from the beginning of skin incision until surgical closure, which could reflect the proficiency of the operators for these two different techniques as well as risk of infection.

#### *The Length of Incision*

The length of incision was measured by the graduated scale. It can reflect the degree of surgical trauma.

#### *The Amount of Operative Blood Loss*

The amount of operative blood loss was the sum of the amount of blood from the suction device and the amount of blood on the gauze. Similar to the length of incision, it can reflect the degree of surgical trauma.

#### *Hospitalization Time and Hospitalization Cost*

We recorded the hospitalization time. The hospitalization time began from the admission of the patient to the end of discharge. Discharge standard: the incision healed normally, the patient returned to normal eating and defecation, the internal medicine disease was stable and did not aggravate, and there was no obvious discomfort in the lower ground activity. We recorded the hospitalization cost, including treatment cost, examination cost, operation cost, and prosthesis cost. Extension of hospitalization time increases the hospitalization cost.

#### *Follow‐up*

All patients were followed up regularly after their operation for at least 12 months. X‐ray films of the pelvis and the injured hip joint were reexamined. The deformities of the affected limb were observed. The position of the total hip prosthesis was observed, considering, for example, whether the implant was stable, whether the prosthesis was loose, whether there was infection, whether there was a fracture around the prosthesis, and whether there was dislocation. The prosthesis abduction angle and the anteversion angle of the acetabulum were observed. The ideal range of the acetabular prosthesis abduction angle is 40° to 50°, and the ideal range of the anteversion angle is 10° to 25°^15^. Among them, the anteversion angle is the most important; anterior dislocation can easily occur when the angle is too large, and posterior dislocation can easily occur when the angle is too small. Whether there were complications such as venous thrombosis of lower extremities was investigated. At the same time, the functional recovery of the hip joint on the injured side was observed.

#### *Visual Analogue Score*

The degree of hip joint pain was evaluated by VAS score. The degree of hip pain was evaluated at 1 week, 1 month, 3 months, 6 months, and 12 months after the operation. According to the VAS system, the degree of hip joint pain was evaluated in all patients. The method involves using the visual analogue graduated scale. One side of the graduated scale was turned back to the patient and the patient was asked to mark the appropriate position on the graduated scale that represents the degree of pain. The score was evaluated according to the patient's mark. The score criteria were as follows: no pain: 0; mild pain, tolerable, not affecting sleep: 1 to 3; moderate pain, mild affecting sleep, still tolerable: 4 to 6; severe pain, unbearable pain, pain resulting in inability to sleep or waking up from sleep: 7 to 10.

#### *Harris Hip Score*

The recovery of hip joint function after surgery was evaluated by Harris score^16^. The hip joint function was evaluated at 1 week, 1 month, 3 months, 6 months, and 12 months after operation. The hip joint function of all patients was evaluated according to Harris score. The score includes four aspects: pain, function, degree of deformity, and range of motion of the joint. The score standard was as follows: excellent: 90 to 100; good: 80 to 89; pass: 70 to 79; poor: <70.

### *Statistical Analysis*

Statistical software IBM SPSS 20.0 (International Business Machines Corporation, Armonk, New York, USA) was used for statistical analysis. For categorical variables, the data between groups were compared using the χ^2^‐test and Fisher's exact test. For quantitative variables, the data between groups were expressed as mean ± SD, and compared by *t*‐test or rank sum test for statistical analysis. A value of *P* < 0.05 indicated a statistically significant difference.

## Results

### *General Information of Participants*

There were 55 patients in the SuperPATH group, including 27 men and 28 women, aged 65 to 75 years, with an average age of 69.03 years. There were 55 patients in the conventional group, including 25 men and 30 women, aged 65 to 75 years, with an average age of 70.13 years. Comparison of the general information of participants between the two groups is shown in Table 1. There were no statistically statistical differences in gender, age, injury side, and fracture type between the two groups, and they were comparable (*P* > 0.05).

**Table 1:** Comparison of the general information of participants between the two groups

Groups	The number of cases	Age (years)	Gender (cases)		Injury side (cases)		Garden classification (cases)	
			Male	Female	Left	Right	III	IV
SuperPATH group	55	69.03±3.01	27	28	22	33	30	25
Conventional group	55	70.13±3.35	25	30	24	31	29	26
*P*‐value		0.091*		0.849*		0.847*		1.000*

* Not statistically significant.

### *Perioperative Outcomes*

Comparison of perioperative outcomes between the two groups is shown in Table 2. Perioperative outcomes include: the operation time, the length of incision, the amount of operative blood loss, the hospitalization time, and the hospitalization cost.

**Table 2:** Comparison of perioperative outcomes between the two groups (mean ± standard deviations)

Groups	Number of cases	Operation time (min)	Length of incision (cm)	Amount of operative blood loss (mL)	Hospitalization time (days)	Hospitalization cost (dollars)
SuperPATH group	55	108.58 ± 15.87	6.65 ± 1.53	147.51 ± 28.84	10.05 ± 2.52	6871.78 ± 141.63
Conventional group	55	102.51 ± 19.61	17.08 ± 1.40	170.22 ± 25.34	13.36 ± 3.39	7791.09 ± 184.88
*P*‐value		0.077	<0.001*	<0.001*	<0.001*	<0.001*

* Statistically significant.

#### *The Operation Time*

The operation time was 108.58 ± 15.87 min in the SuperPATH group and 102.51 ± 19.61 min in the conventional group. There was no significant difference between the two groups (*P* > 0.05). Our results suggest that this new technique would not prolong the operation time and increase risk of infection.

#### *The Length of Incision*

The length of incision was 6.65 ± 1.53 cm in the SuperPATH group and 17.08 ± 1.40 cm in the conventional group. Compared with the conventional group, the length of incision in the SuperPATH group was reduced by 61.07%. There was a significant difference between the two groups (*P* < 0.05).

#### *The Amount of Operative Blood Loss*

The amount of operative blood loss was 147.51 ± 28.84 mL in the SuperPATH group and 170.22 ± 25.34 mL in the conventional group. Compared with the conventional group, the amount of operative blood loss in the SuperPATH group was reduced by 13.34%. There was significant difference between the two groups (*P* < 0.05).

#### *Hospitalization Time and Hospitalization Cost*

The hospitalization time was 10.05 ± 2.52 days for the SuperPATH group and 13.36 ± 3.39 days for the conventional group. Compared with the conventional group, the hospitalization time in the SuperPATH group was shortened by 24.78%. There was a significant difference between the two groups (*P* < 0.05).

The hospitalization cost was 6871.78 ± 141.63 dollars for the SuperPATH group and 7791.09 ± 184.88 dollars for the conventional group. Compared with the conventional group, the hospitalization cost in the SuperPATH group was reduced by 11.80%. There was a significant difference between the two groups (*P* < 0.05).

### *Postoperative Outcomes*

#### *Follow‐up*

**Figure 2:**
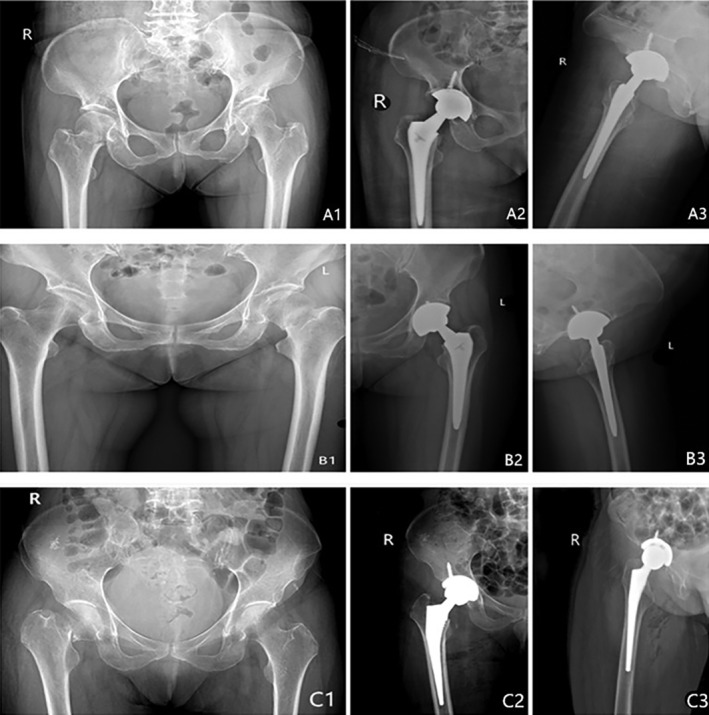
Preoperative X‐rays (A1, B1, C1) of femoral neck fractures from 3 patients ((A) 70‐year‐old woman; (B) 67‐year‐old woman; (C) 71‐ year‐old woman) who had been treated by SuperPATH minimally invasive approach total hip arthroplasty (A2, A3, B2, B3) and conventional posterolateral approach artificial total hip arthroplasty (C2, C3).

All patients were followed up for at least 12 months. No hip varus or valgus deformity was found on the injured side of all patients. The prosthesis abduction and anteversion angles of all patients were within the ideal range. During the follow‐up period, there were no complications such as infection, lower extremity venous thrombosis, prosthesis loosening, periprosthetic fracture, or dislocation. Typical cases are shown in Fig. 2.

#### *Visual Analogue Score*

All patients were evaluated for the degree of hip pain at 1 week, 1 month, 3 months, 6 months, and 12 months after the operation. Comparison of the VAS score between the two groups is shown in Table 3.

**Table 3:** Comparison of the visual analog score score between the two groups (mean ± standard deviation)

Groups	The number of cases	1 week after operation	1 month after operation	3 months after operation	6 months after operation	12 months after operation
SuperPATH group	55	4.45 ± 0.94	1.79 ± 0.69	1.20 ± 0.58	0.86 ± 0.53	0.71 ± 0.45
Conventional group	55	4.89 ± 0.79	1.92 ± 0.65	1.32 ± 0.67	0.91 ± 0.50	0.81 ± 0.38
*P*‐value		<0.05*	0.317	0.325	0.606	0.236

* Statistically significant.

The VAS score was 4.45 ± 0.94 in the SuperPATH group and 4.89 ± 0.79 in the conventional group at 1 week after the operation. Compared with the conventional group, the VAS score in the SuperPATH group was better than that in conventional group at 1 week after the operation. There was a significant difference between the two groups (*P* < 0.05). There was no significant difference in the VAS score between the two groups at 1 month, 3 months, 6 months, and 12 months after the operation (*P* > 0.05).

### *Harris Hip Score*

All patients were evaluated for hip joint function at 1 week, 1 month, 3 months, 6 months, and 12 months after the operation. Comparison of the Harris hip score between the two groups is shown in Table 4.

**Table 4:** Comparison of the Harris hip score between the two groups (mean ± standard deviation)

Groups	The number of cases	1 week after operation	1 month after operation	3 months after operation	6 months after operation	12 months after operation
Super PATH group	55	75.36 ± 3.36	80.25 ± 3.09	81.13 ± 3.84	83.27 ± 5.12	86.65 ± 5.46
Conventional group	55	68.80 ± 3.25	77.35 ± 3.77	80.33 ± 4.33	82.96 ± 4.38	86.27 ± 5.27
*P*‐value		<0.001*	<0.001*	0.307	0.734	0.710

* Statistically significant.

The Harris score was 75.36 ± 3.36 and 80.25 ± 3.09 in the SuperPATH group and 68.80 ± 3.25 and 77.35 ± 3.77 in the conventional group at 1 week and 1 month after surgery, respectively. Compared with the conventional group, the Harris score in the SuperPATH group was better than that in the conventional group at 1 week and 1 month after the operation. There was a significant difference between the two groups (*P* < 0.05). There was no significant difference in Harris score between the two groups at 3 months, 6 months, and 12 months after the operation (*P* > 0.05).

## Discussion

Femoral neck fractures often occur in the middle‐aged and the elderly, and are often associated with osteoporosis and other medical diseases; falls and twisting can lead to fractures. Following traffic accidents and high‐energy trauma, young adults can also suffer femoral neck fractures. There are different treatment schemes depending on the age of the patient and the type of femoral neck fracture^17^. In clinical practice, the most suitable treatment method is generally selected through the evaluation of patients’ overall health status, age, fracture classification, and other aspects. Elderly patients with femoral neck fractures are often characterized by poor general health, often accompanied by a variety of complications. Conservative treatment requires long‐term bed rest, so that patients are prone to pulmonary infection, pressure sores, deep venous thrombosis, and other serious complications, which can be life‐threatening. For elderly patients with femoral neck fractures, in the absence of absolute surgical contraindications, most of them advocate total hip arthroplasty for treatment^18^. Through the reconstruction of hip joint function, total hip arthroplasty can relieve joint pain, correct deformity, restore hip joint motor function, and effectively improve the prognosis of patients^19^. It is the main method used to treat femoral neck fractures in the elderly. The choice of surgical approach for total hip arthroplasty is closely related to the recovery of hip joint function, the stability of the artificial prosthesis, and the risk of dislocation. The destruction of muscle and soft tissue using the surgical approach can affect the stability of the hip joint and increase the risk of dislocation. Therefore, reducing the soft tissue injury without affecting the curative effect of artificial prosthesis implantation is critical to the success of the operation and an important factor in determining the ideal surgical approach. At present, the posterolateral approach is the most widely used approach in conventional total hip arthroplasty. This approach is risks damaging the sciatic nerve. In addition, this approach requires resection of the posterior external rotator muscle and the articular capsule of the hip joint, which causes excessive muscle and soft tissue damage, and increases the degree of surgical trauma and the amount of bleeding. At the same time, the resection of the posterior circumflex muscle groups of the hip joint will affect the abduction strength of the affected limb, which is disadvantageous to the early functional recovery of the patient. It is especially dangerous for elderly patients with poor muscle strength and physical coordination. It prolongs the rehabilitation time, increases the hospitalization time, and increases the hospitalization cost. It will have a great impact on the financial and nursing burden of patients and families.

In recent years, with the improvement of the minimally invasive approach for total hip arthroplasty and the accumulation of experience, minimally invasive total hip arthroplasty has been accepted by more and more surgeons^20^. The significance of minimally invasive total hip arthroplasty is that the effect of artificial prosthesis implantation is not affected; at the same time, the skin incision is small, and the hip joint is exposed through the muscle space channel, to minimize the intraoperative local soft tissue injury, reduce intraoperative and postoperative bleeding, and accelerate the functional recovery of the hip joint^21^. At present, the commonly used minimally invasive surgical approach for total hip arthroplasty has its own advantages and disadvantages. The comparison of different surgical approaches has been the focus of debate in recent years^22^. For example, the DAA approach does not involve cutting off the periarticular muscles but preserves the periarticular muscles. However, it is easy to damage the lateral femoral cutaneous nerve, resulting in postoperative thigh numbness and pain^23^. The minimally invasive anterolateral approach is also a muscle space approach, does not damage muscles and other soft tissues, and preserves the joint capsule. The deficiency lies in the insufficient exposure of the incision, which requires adduction and extension to deal with the femur, which can easily lead to fracture of the proximal femur. When placing the acetabular cup, if the anterior inclination angle is too large, postoperative anterior dislocation can occur. The advantages and disadvantages of the minimally invasive posterolateral approach were found to be similar to those of the posterior approach. The operative field was exposed clearly and the placement of the prosthesis was accurate. However, the external circumflex muscle group and joint capsule were seriously damaged, resulting in poor joint stability, and the postoperative recovery time was longer^24^. To sum up, the visual field of the small incision approach is limited, and it is relatively difficult to place prostheses. As a result, it may increase the likelihood of nerve injury, femoral fracture, and poor prosthesis position. The real minimally invasive surgical technique of total hip arthroplasty not only involves a small incision but also needs to be a technique that achieves the best surgical results with minimal invasion and physiological interference. The SuperPATH approach combines the SuperCap approach with the PATH approach^25^, ^26^. The approach involves a small incision and entry is through the gap between the gluteus minimus and piriformis, exposing the surgical field with the special tools. The acetabulum is treated by percutaneous puncture channel and the femur is treated *in situ*. There is no need to cut off the muscles so that the soft tissue and joint capsule around the hip joint can be protected. The stability of the hip joint is maintained and the risk of dislocation is reduced. The application of the SuperPATH minimally invasive approach to total hip arthroplasty for the treatment of femoral neck fractures in the elderly can obtain the best surgical effect with minimal invasion and physiological interference. The patients can suffer minor surgical injuries.

The main results of this control study are summarized as follows. First, in the analysis of the length of incision, the amount of blood loss, the hospitalization time, and the hospitalization cost between the two groups, the results for the SuperPATH group were better than for the conventional group. There was significant difference between the two groups (*P* < 0.05). Second, at 1 week after the operation between the two groups, the VAS score results were better for the SuperPATH group than for the conventional group. There was significant difference between the two groups (*P* < 0.05). Third, for the Harris score at 1 week and 1 month after the operation, the results of the SuperPATH group were better than for the routine group. There was significant difference between the two groups (*P* < 0.05). Fourth, all patients in the SuperPATH group were followed up and reexamined by X‐ray. The prosthesis abduction angle and anteversion angle of all patients were within the ideal range. No hip varus or valgus deformity was found on the injured side of all patients. During the follow‐up period, there were no complications such as infection, lower extremity venous thrombosis, prosthesis loosening, periprosthetic fracture, and dislocation. The prosthesis material of the SuperPATH approach is different from that of the conventional posterolateral approach. Whether there are differences in joint function, wear degree, and service life of the prosthesis still needs to be followed up and observed for a longer period of time. It will be supplemented and perfected in further research in the future. The SuperPATH minimally invasive approach to total hip arthroplasty is a new surgical method for the treatment of femoral neck fractures in the elderly. The results show that the new surgical method can effectively shorten the length of incision, reduce the amount of blood loss, and reduce the surgical trauma, as well as effectively shorten the hospitalization time and reduce the hospitalization cost. The VAS score of hip joint pain in the short term after the operation was better than that for the conventional posterolateral approach. Effective relief of postoperative pain is beneficial to the early rehabilitation of patients. The Harris score of hip joint function in the short term after surgery was better than that for the conventional posterolateral approach. The function of the hip joint recovered quickly after surgery. This depends on the SuperPATH approach not cutting off the external rotator muscle groups, so that the function of the hip joint is better preserved. Compared with the conventional surgical method, patients can go to the ground earlier, resume physical activity sooner, commence ehabilitation exercises earlier, and be discharged from hospital earlier. It can significantly improve the curative effect.

The SuperPATH minimally invasive approach to total hip arthroplasty for the treatment of femoral neck fractures in the elderly has a wide range of indications, and is suitable for patients who are candidates for the conventional posterolateral approach. Relative contraindications include: (i) muscular patients or obese patients with BMI > 30 kg/m^2^; (ii) patients with hip ankylosis or fusion; (iii) patients with extremely severe osteoporosis; and (iv) patients with severe hip dysplasia (Crowe type III or above). Absolute contraindications include: (i) patients with bone destruction at the proximal end of the femur (e.g. a bone tumor); and (ii) patients with a history of hip surgery before fracture. This surgical method has the following advantages in application. First, the surgical instruments are more precise and standardized, the instruments can be mastered and used quickly. Second, the main operation of the the SuperPATH approach can be completed by a chief surgeon and two assistants. Use of the self‐supporting retractor reduces the unnecessary risk of infection and ensures the safety of the operation. Third, the operation is minimally invasive and the surgical trauma is minimal. It enables rapid recovery of patients after surgery. The incision length of the SuperPATH approach is 6 to 8 cm, which is half of that for conventional hip arthroplasty. The SuperPATH approach does not require cutting off the external circumflex muscle groups, and almost all the muscle function around the hip joint and the complete joint capsule are preserved. There was no excessive soft tissue injury. The amount of surgical bleeding and the degree of surgical trauma were reduced. The femoral head was resected *in situ* during the operation to avoid traction of the lower extremities. Traction injury to soft tissue such as ligaments was reduced and the accurate eccentricity was restored. It is beneficial for early rehabilitation of patients after operation and return to exercise. To a certain extent, it also satisfies the concept of ERAS. Finally, there is no special postural restriction after the operation. After conventional hip arthroplasty, the affected limb is required to lie flat in the abduction neutral position, and the flexion of the hip and knee is limited. In this way, the rehabilitation process has a heavy nursing burden. There is no special postural restriction in patients treated with the SuperPATH approach. At the same time, it is more conducive to early postoperative hip joint active exercise and lower limb strength training. It is more beneficial to the recovery of hip joint function.

Attention should be attention to the following in the treatment of senile femoral neck fractures with the SuperPATH minimally invasive approach to total hip arthroplasty. First, when penetrating the femoral stalk, it should be close to the lateral cortex of the femur, and the proximal medullary cavity needs to be scraped to the cortical bone to achieve better results. Second, the depth of insertion should be checked with an open file handle and the depth of the top of the open file measured relative to the tip of the greater rotor, which is usually 15 to 25 mm. Of course, depending on the anatomy of the patient and the difference in the length of the lower limb before surgery, the medullary cavity probe can also be used to confirm the depth of insertion. Third, the femoral head can be removed by rotating the cross thread needles. When it is difficult to remove, it can be taken out by chiseling into small pieces. Fourth, the appropriate size acetabular file is placed through the main incision and the rod of the acetabular file is inserted through the casing. When grinding the acetabulum, attention should be paid to the leading edge of the acetabulum. First, the acetabular cup fossa should be ground with a small acetabular file through the casing passage, and then the acetabulum should be ground in turn. Fifth, the acetabular cup should be placed with attention to the abduction angle and the anteversion angle. Finally, most important of all, total hip arthroplasty should be performed with a focus on surgical safety. The indications and contraindications of the minimally invasive surgical approach should be understood. Incision minimization should not be pursued blindly. If the operation becomes difficult, the incision should be extended immediately and the method should be changed to the conventional surgical method.

To sum up, the SuperPATH minimally invasive approach to total hip arthroplasty is an ideal surgical method for the treatment of femoral neck fractures in the elderly. It overcomes the shortcomings of traditional total hip arthroplasty, and improves the speed of recovery and gets patients out of bed earlier. This surgical method of operation is relatively simple. It helps to reduce the amount of operative blood loss, reduces surgical trauma, and reduces the hospitalization time of patients. It lowers the hospitalization cost, reduces the burden of patients, and improves the function of the hip joint soon after surgery. It is in line with the modern concept of ERAS. However, there are still some shortcomings in this study. The number of cases is relatively small, and there is a lack of long‐term follow‐up observation. It will be supplemented and perfected in further research in the future.
